# Frequency of BRAF V600E mutations in 969 central nervous system neoplasms

**DOI:** 10.1186/s13000-016-0506-2

**Published:** 2016-06-27

**Authors:** Felix Behling, Alonso Barrantes-Freer, Marco Skardelly, Maike Nieser, Arne Christians, Florian Stockhammer, Veit Rohde, Marcos Tatagiba, Christian Hartmann, Christine Stadelmann, Jens Schittenhelm

**Affiliations:** Department of Neurosurgery, Eberhard-Karls University, Hoppe-Seyler Street 3, 72076 Tübingen, Germany; Department of Neuropathology, Georg-August University, Robert-Koch-Street 40, 37075 Göttingen, Germany; Department of Pathology, Eberhard-Karls University, Liebermeisterstr. 8, 72076 Tübingen, Germany; Department of Neuropathology, Hannover Medical School, Carl-Neuberg Street 1, 30625 Hannover, Germany; Department of Neurosurgery, Städtisches Klinikum Dresden-Friedrichstadt, Friedrichstr. 41, 01067 Dresden, Germany; Department of Neurosurgery, Georg-August University, Robert-Koch-Street 40, 37075 Göttingen, Germany; Department of Neuropathology, Eberhard-Karls University, Calwer Str. 3, 72076 Tübingen, Germany

**Keywords:** BRAF V600E mutation, Tissue microarray, Brain tumor, Cerebral metastases, Epithelioid glioblastoma, Glioblastoma, Gliosarcoma, Rhabdoid meningioma

## Abstract

**Background:**

Treatment options for oncological diseases have been enhanced by the advent of targeted therapies. The point mutation of the BRAF gene at codon 600 (BRAF V600E) is found in several tumor entities and can be approached with selective inhibitory antibodies. The BRAF inhibitor vemurafenib has demonstrated clinical efficacy in patients with BRAF V600E-mutant melanoma brain metastases and in other cancer diseases. Therefore the BRAF V600E mutation is a highly interesting oncological target in brain tumors.

**Methods:**

This study assesses the BRAF V600E mutation status in 969 intracranial neoplasms using a tissue microarray method and immunohistochemical staining with the mutation-specific VE-1 antibody, followed by sequencing of positively stained cases.

**Results:**

Out of 784 primary brain tumors seven cases with a BRAF V600E mutation were detected (7/784, 1 %). Six of these cases were neuroepithelial tumors (6/667, 1 %) encompassing 2 astrocytomas WHO grade II (2/42, 5 %), 1 gliosarcoma WHO grade IV (1/75, 1 %) and 3 glioblastomas WHO grade IV (3/312, 1 %). Interestingly, all three mutant glioblastomas showed epithelioid histopathological features. Patients with V600E mutated astrocytic tumors were significantly younger (mean age 15.3 years) than wildtype cases (58.2 years). Among three rhabdoid meningiomas, one case was mutated (1/3) while all other grade I-III meningiomas (1/116, 1 %) and all fifty vestibular schwannomas analyzed were of wildtype status. The vast majority of the BRAF V600E mutations were found in cerebral metastases of malignant melanomas and carcinomas (29/135, 22 %), with false-positive staining found in four breast cancer cases and two non-small-cell lung carcinoma (NSCLC) samples.

**Conclusions:**

Our data suggest routine screening for BRAF V600E mutations for glioblastomas WHO grade IV below the age of 30, especially in glioblastomas with epithelioid features and in all rhabdoid meningiomas WHO grade III. For colorectal carcinoma, thyroid cancer, malignant melanoma and gliomas BRAF V600E immunostaining is sufficient for screening purposes. We also recommend routine immunohistochemical staining followed by sequencing validation in rare CNS metastases or metastases of unknown primary.

Immunohistochemical analysis using mutation-specific antibodies on tissue microarrays is a feasible, time- and cost-efficient approach to high-throughput screening for specific mutations in large tumor series but sequencing validation is necessary in unexpected cases.

**Electronic supplementary material:**

The online version of this article (doi:10.1186/s13000-016-0506-2) contains supplementary material, which is available to authorized users.

## Background

With the advent of deeper insights into the development and molecular identity of tumors, targeted therapies have become increasingly interesting and have shown efficacy in several tumor entities [1, 2]. One of the best-studied targets is the proto-oncogene B-Raf (BRAF) that encodes a serine/threonine protein kinase of the RAS-RAF-MEK-ERK-MAP kinase pathway. This highly regulated pathway controls cell growth and can be disrupted by BRAF alterations, which transform the BRAF kinase into a constitutively activated form resulting in excessive cell proliferation and thus enabling tumor growth [3]. Especially the BRAF V600E mutation has been described in up to 7 % of human cancers [4]. This specific mutation causes an exchange of valine for glutamine at position 600 of the amino acid sequence of the protein kinase. It is a well-characterized target in malignant melanoma and can be found in approximately 66 % of primary cases [4]. Direct targeting with B-Raf kinase inhibitors such as vemurafenib or dabrafenib is an effective new treatment option and has been approved for advanced malignant melanomas harboring the BRAF V600E mutation [5]. Recently, a mutation-specific monoclonal antibody (VE-1) for the BRAF V600E mutation has been developed [6] and successfully validated in malignant melanoma, colorectal and papillary thyroid cancer as well as non-small-cell lung carcinoma (NSCLC), pleomorphic xanthoastrocytomas (PXA) [7–11].

In some glioma types the antibody is even considered superior to sequencing [12]. Overall, BRAF mutations play an important role in neurooncology. An analysis of 885 brain metastases revealed mutations in metastases of melanoma (55.3 %), ovarian (6.7 %), colorectal (5.5 %), lung (0.3 %) and thyroid (33.3 %) cancer [13]. Interestingly, the frequency of BRAF mutations in primary lung cancer is higher – an overview reported that 36 out of 883 NSCLCs had BRAF V600E mutations [14]. Subsequent studies confirmed the lower frequency of V600E mutations in NSCLC brain metastases, indicating that frequencies of V600E mutated metastases in the brain might differ from those in primary locations [15]. Approximately 10 % of all colorectal cancer specimens carry the V600E mutation, but unfortunately this tumor type does not respond well to inhibitor treatment [16]. In papillary thyroid carcinomas the mutation was reported to be present in about 45 % [17, 18] and there is evidence that it has a negative prognostic impact [19]. Both entities occasionally metastasize to the brain. Data on mutation frequency in these brain metastases is still limited.

BRAF signal alterations are also involved in primary brain tumors. In 2008 a tandem-duplication at 7q34 was identified, resulting in fusion of the previously uncharacterized gene KIAA1549 and the BRAF gene to create a novel fusion oncogene [20]. While this fusion transcript is relatively specific to one pediatric brain tumor, the pilocytic astrocytoma (up to 70 % of the cases), a subsequent study of 1320 nervous system tumors revealed exceptionally high rates of BRAF V600E mutations in pleomorphic xanthoastrocytomas (PXA) WHO grade II (66 %) and III (65 %) as well as gangliogliomas WHO grade I (18 %) and III (50 %). Moreover, the BRAF V600E mutation was also found in 9 % of pilocytic astrocytomas, mutually exclusive with the KIAA1549-BRAF fusion and was associated with extracerebellar location [21]. In PXA, recent data indicate a favorable course for V600E mutated tumors [22], while in ganglioglioma and diencephalic tumors it is considered a negative prognostic factor [23, 24]. In pediatric diffusely infiltrating astrocytomas BRAF V600E mutation frequencies between 17 – 29 % have been reported in smaller studies [25–28]. The high V600E frequency rates in pediatric brain tumors were recently confirmed in a larger independent cohort [29].

As a consequence of these findings and the good outcome in the therapy of malignant melanoma, several reports of salvage kinase inhibitor treatment in V600E-mutated, advanced cerebral tumor entities have been published. One case of an anaplastic ganglioglioma of the brainstem showed decrease in size and enhancement on magnetic resonance imaging (MRI) controls after receiving vemurafenib combined with vinblastine [30]. Bautista et al. presented 3 pediatric cases with BRAF mutated malignant gliomas. One out of 2 anaplastic gangliogliomas and one anaplastic astrocytoma responded to vemurafenib treatment [31]. In a case series of 4 advanced BRAF V600E mutated PXAs WHO grade II vemurafenib was applied as a salvage therapy. The treatment resulted in disease stabilization in two cases and partial response of one patient [32]. Another clinical response was observed in a patient with a meningeal PXA with anaplastic features and a BRAF V600E mutation [33]. Tumor regression under vemurafenib was also reported in a case of advanced pilomyxoid astrocytoma [34].

Even though unmasked as a rarity by several studies, the BRAF V600E mutation in glioblastomas revealed interesting aspects. Epithelioid glioblastomas may harbor a BRAF V600E mutation in 50 % of the cases [35]. One case of a pediatric glioblastoma with focal epithelioid features has been reported where vemurafenib was applied after tumor recurrence. Surprisingly, the 9-year old boy showed regression of the enhancement of the V600E-mutated tumor on subsequent MRIs [36]. Another recent case of a quick recurring epithelioid glioblastoma underwent a second resection with adjuvant vemurafenib application after BRAF V600E mutation was detected. The last report stated that the patient was tumor free for 21 months [37]. Based on promising results from in vitro application of BRAF V600E inhibitory substances, further evaluation of BRAF V600E treatment options in malignant astrocytoma was suggested [38].

Intracranial neoplasms as collected in this study are the typical tumor entities encountered in a neurooncological tumor board. Cases with primary central nervous system (CNS) tumors and metastases in advanced stages, where established therapies are exhausted, are regularly discussed for a possible inhibitory antibody treatment against BRAF V600E mutated tumor cells. Treatment is established and approved for metastasized malignant melanoma and, as stated above, numerous case reports suggest efficacy in other advanced neurooncological tumor types.

The primary goal of this study was to assess the usefulness of the VE-1 antibody to detect V600E mutated samples as a first step in neurooncological tumor routine work up. Because of the expected differences in frequencies, data of primary brain tumors and metastases are presented separately.

A special emphasis was placed on brain metastases, meningiomas, gliosarcomas and vestibular schwannomas that were underrepresented in previous studies. Our aim is to establish an age and histology-dependent rationale regarding the choice of neurooncological tumor types that should undergo routine BRAF V600E mutation screening, thus allowing the possibility of targeted treatment with kinase inhibitors and to determine the efficiency of the BRAF mutation specific VE-1 antibody to detect the V600E hotspot mutation successfully in archival specimens.

## Methods

In total 969 brain tumors were analyzed including 667 neuroepithelial tumors, 117 meningiomas, 135 metastases and 50 vestibular schwannomas. The detailed histopathological entities are listed in Table 1. Histological diagnosis and grading for each tumor sample were performed according to the current WHO classification system by at least two, or in most cases three board-certified neuropathologists (CH, CS, JS). Basic clinical characteristics of the assessed cases (age at diagnosis and gender) were collected and statistically analyzed (ANOVA followed by student t for correlation) with the software JMP® Version 10. (SAS Software, Cary, NC, USA). Archived paraffin embedded tissue samples from the Institutes of Neuropathology Tübingen and Göttingen were processed into tissue microarray (TMA) blocks.

**Table 1:** Frequency of BRAF V600E mutations in 969 CNS tumors

	n BRAF V600E/total	%	Mean age
Neuroepithelial tumors:	6/667	0.90	54
Astrocytoma (WHO grade II)	2/42	4.76	44
Oligoastrocytoma (WHO grade II)	0/12	0.00	41
Oligodendroglioma (WHO grade II)	0/19	0.00	41
Anaplastic astrocytoma (WHO grade III)	0/36	0.00	44
Anaplastic oligoastrocytoma (WHO grade III)	0/15	0.00	39
Anaplastic oligodendroglioma (WHO grade III)	0/40	0.00	51
Glioblastoma (WHO grade IV)	3/312	0.96	60
Gliosarcoma (WHO grade IV)	1/75	1.33	62
Subependymoma (WHO grade I)	0/5	0.00	53
Myxopapillary ependymoma (WHO grade I)	0/16	0.00	38
Ependymoma (WHO grade II)	0/46	0.00	50
Anaplastic ependymoma (WHO grade III)	0/13	0.00	39
Choroid plexus papilloma (WHO grade I)	0/28	0.00	47
Choroid plexus papilloma (WHO grade II)	0/6	0.00	41
Choroid plexus carcinoma (WHO grade III)	0/2	0.00	43
Cranial nerve tumors:			
Vestibular schwannoma (WHO grade I)	0/50	0.00	44
Meningeal tumors:	1/117	0.85	45
Meningioma (WHO grade I)	0/55	0.00	41
Meningothelial	0/28	0.00	40
Transitional	0/17	0.00	41
Fibrous	0/3	0.00	41
Psammomatous	0/3	0.00	48
Microcystic	0/2	0.00	45
Not otherwise specified	0/2	0.00	50
Meningioma (WHO grade II)	0/35	0.00	39
Atypical	0/26	0.00	35
Chordoid	0/2	0.00	43
CNS infiltration	0/2	0.00	45
Not otherwise specified	0/5	0.00	59
Meningioma (WHO grade III)	1/27	3.70	60
Anaplastic	0/24	0.00	62
Rhabdoid	1/3	33.33	36
Metastases:	29/135	21.48	57
Melanoma	24/58	41.38	56
NSCLC	0/29	0.00	59
Adeno Ca	0/22	0.00	59
Squamous cell Ca	0/5	0.00	60
Not otherwise specified	0/2	0.00	58
Breast Ca	0/22	0.00	54
Colorectal Ca	2/4	50.00	51
Sarcoma	0/7	0.00	57
Prostate Ca	0/6	0.00	66
Renal clear cell Ca	0/3	0.00	71
Esophageal adeno Ca	1/2	50.00	46
Hepatcellular Ca	1/1	100.00	73
Papillary thyroid Ca	1/1	100.00	74
Parotid acinic cell Ca	0/1	0.00	41
Ovarian Ca	0/1	0.00	45

Hematoxylin and eosin (H&E) stains of each sample were assessed beforehand and tumor areas most suitable for sample cylinder extraction were marked. Representative tumor cores were selected, excluding necrotic tumor areas, inflammation, stroma-rich areas or infiltration borders. The tissue sample size was 1 or 2 mm in diameter and in most cases two donor cylinders were obtained and aligned on recipient blocks using a conventional tissue microarrayer (Beecher Instruments, Sun Prairie, Wisconsin, USA). The newly constructed TMA blocks were cut in 4 μm slices, dried at 80 °C for 15 min and stained immediately. Immunohistochemical staining was performed on a Ventana BenchMark immunostainer (Ventana Medical Systems, Tucson, USA). The Ventana staining procedure included pretreatment with cell conditioner 1 (pH 8) for 72 min (VE-1), followed by incubation with primary antibodies (VE-1 dilution 1:5) at 37 °C for 32 min to mark BRAF V600E mutated tumor cells [6]. The anti-BRAF V600E (VE-1 clone, a kind gift from Dr. Capper, Neuropathology Heidelberg) antibody is a mutation-specific mouse monoclonal antibody that was raised against a synthetic peptide representing the BRAF V600E mutated amino acid sequence from amino acids 596 to 606 (GLATEKSRWSG). The same antibody is commercially available by Ventana Roche (catalogue number 790-4855). Antibody incubation was followed by OptiView HQ Universal Linker for 12 min, incubation with OptiView HRP Multimer for 12 min. Stains were counterstained with one drop of hematoxylin for 4 min. Melanoma samples with validated BRAF V600E mutation served as positive control in each run. The stained TMA slides were microscopically evaluated. BRAF V600E staining was considered positive, when more than 1 % of the visible tumor cells were at least weakly immunoreactive for BRAF V600E. Any type of isolated nuclear staining, weak staining of single interspersed cells, or staining of monocytes/macrophages was scored negative. In 12 cases repeated VE-1 staining on full slides was necessary, because either positive controls included in each batch were not stained sufficiently or a very weak unspecific background staining was present.

All immunopositive samples identified by BRAF V600E immunohistochemistry subsequently underwent Sanger sequencing for V600E mutation status except for melanoma metastases, where the antibody has been validated previously. Tumor deoxyribonucleic acid (DNA) was extracted from selected areas with sufficient tumor content on full slides using the black Prep DNA Mini Kit (Analytik Jena, Germany) according to manufacturer’s instructions. Amplification of BRAF polymerase chain reaction (PCR) products was carried out with 200 ng of genomic DNA and the following PCR primers: BRAF fwd: 5’-TGTAAAACGACGGCCAGTTCATAATGCTTGCTCTGATAGGA-3’ and BRAF rev: 5’-CAGGAAACAGCTATGACCTAGCTCAGCAGCATCTC-3’ (M13-tailed). PCR conditions were performed with an initial denaturation at 95 °C for 5 min, denaturation at 95 °C for 45 s, annealing at 56 °C for 1.15 min and extension at 72 °C for 1 min for 45 cycles followed by final elongation at 72 °C for 7 min. For the PCR amplification of the BRAF fragment the AmpliTaq Gold® DNA polymerase (Applied Biosystems) was used. Subsequent to the PCR amplification PCR products were purified with Agencourt AMPure Beads (Beckman Coulter) following the manufacturer’s instructions. The PCR products were sequenced using a GenomeLab™ GeXP Genetic Analysis System (Beckman Coulter).

## Results

**Fig. 1:**
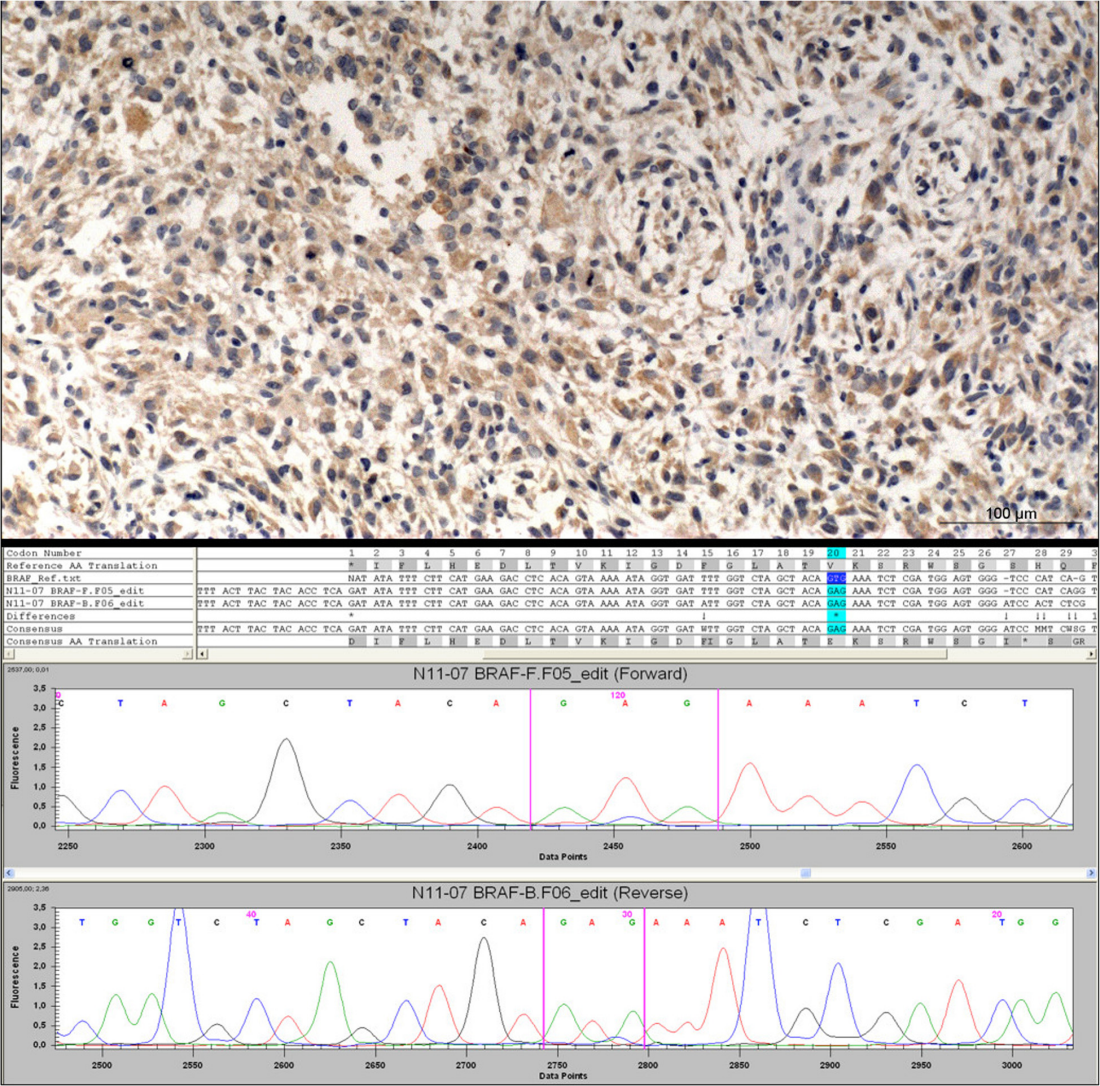
BRAF V600E mutation analysis via immunohistochemistry and Sanger sequencing: BRAF V600E immunopositive high-grade glioma with typical homogenous perinuclear staining of tumor cells (upper panel). Sanger sequencing of a BRAF V600E mutated sample of a rhabdoid meningioma (lower panel)

Thirty-six out of 969 (4 %) analyzed central nervous system neoplasms were immunohistochemically positive for the BRAF V600E mutation (Table 1). The majority of immunopositive cases showed moderate to strong cytoplasmic staining of almost all tumor cells while endothelia as well as inflammatory and other non-tumorous cells were spared (Fig. 1). In neuroepithelial tumors, positive antibody staining was present in six out of 667 (<1 %) tumors. When present, the necrotic and perinecrotic tumor areas in immunopositive cases had a marked reduction of VE-1 staining. Direct sequencing for the BRAF V600E mutation in isolated tumor DNA was used for confirmation of the mutation in these tumors (Fig. 1). The mutated samples included two diffuse astrocytomas WHO grade II (two out of 42 samples (4.76 %)) and 4 WHO grade IV tumors (three out of 312 glioblastomas (0.96 %) and 1 out of 75 gliosarcomas (1.33 %)). According to a retrospective analysis of the samples and patient characteristics, all three glioblastomas showed epithelioid features and two were exceptionally young compared to the average age of glioblastoma patients (8, 22 and 70 years of age at time of diagnosis). The patient with the mutated gliosarcoma was 27 years old at the time of diagnosis (Table 2). None of the wildtype glioblastomas exhibited epithelioid features in the histology. All WHO grade III gliomas were immunonegative for VE-1 (36 anaplastic astrocytomas, 15 anaplastic oligoastrocytomas, 40 anaplastic oligodendrogliomas and 13 anaplastic ependymomas). The two patients with mutated diffuse astrocytomas WHO grade II were 16 and 17 years old, while all other low grade gliomas in our cohort were immunonegative and had an average age of 46 years (12 oligoastrocytomas, 19 oligodendrogliomas and 46 ependymomas). The mean age of wildtype astrocytic tumors (astrocytomas WHO grade II, anaplastic astrocytomas WHO grade III, glioblastomas and gliosarcoma WHO grade IV) was 58.2 years (95 % confidence interval: 56.8-59.5) while the mean age in BRAF V600E mutated cases was 15.3 years (95 % CI: 3.6-27.1) and thus significantly younger (student t-test, *p* < 0.0001). Additionally, 36 choroid plexus tumors as well as 50 vestibular schwannomas were analyzed. None of them showed evidence of a BRAF V600E mutation (Table 1).

**Table 2:** Age of BRAF V600E mutated neuroepithelial and meningeal tumors

Histopathology	Age	BRAF V600E Mt
Epithelioid glioblastoma (WHO grade IV)	8	Yes
Epithelioid glioblastoma (WHO grade IV)	70	Yes
Epithelioid glioblastoma (WHO grade IV)	22	Yes
Gliosarcoma (WHO grade IV)	27	Yes
Diffuse astrocytoma (WHO grade II)	16	Yes
Diffuse astrocytoma (WHO grade II)	17	Yes
Rhabdoid meningioma (WHO grade III)	15	Yes

We also analyzed a broad spectrum of meningiomas of different subtypes. All WHO grade I and II meningiomas were immunonegative (0/55 and 0/35, respectively, Table 1) while one out of three rhabdoid meningiomas WHO grade III was mutated, which was confirmed by direct Sanger sequencing of the relevant case. The patient was 15 years of age at the time of diagnosis while the other two VE-1 negative rhabdoid meningioma cases were 45 and 47 years old. Extensive histopathological re-analysis of this case confirmed the diagnosis of a rhabdoid meningioma. All other high-grade meningiomas (24 anaplastic meningiomas WHO grade III) were immunonegative and were from patients of older age, the youngest patient being 38 years old at the time of diagnosis.

Overall, one quarter of the cerebral metastases of this study were immunopositive for the BRAF V600E mutation (29/135, 21.48 %). As expected from previous data, melanoma metastases showed a highe mutation rate with 41.38 % (24/58 cases). One case of a papillary thyroid carcinoma and one hepatocellular carcinoma (HCC) cerebral metastasis were also VE-1 positive and sequencing confirmed the V600E mutation. One out of two esophageal adenocarcinoma metastases was also immunopositive (50 %), as well as two out of four colorectal carcinoma metastases (50 %). Again, in these cases sequencing confirmed the BRAF V600E mutation. Cerebral metastases of different sarcoma types (0/7), prostate cancer (0/6), renal clear cell carcinomas (0/3) as well as one ovarian carcinoma and one parotid acinic cell carcinoma were all VE-1 negative. Out of 22 breast cancer metastases, four cases showed weak to moderate immunohistochemical staining for VE-1. However, Sanger sequencing revealed these breast cancer metastases to be non-mutated for BRAF at codon 600 (0/22). There were also two NSCLC adenocarcinoma metastases that were weakly immunopositive for VE-1, but were identified as wildtype after sequencing. All 29 NSCLC metastases remained non-mutated. In contrast to these six false positive metastases, immunopositivity and Sanger sequencing were always concordant in all neuroepithelial tumor samples except for one gliosarcoma with very weak and diffuse unspecific VE-1 immunoreactivity in the mesenchymal tumor component.

## Discussion

The BRAF V600E mutation is rarely found in neuroepithelial tumors and its exact role in this context is unclear. Apart from one large study [21] a detailed clinical description and frequency of BRAF mutated cases has not been performed. Additionally, some intracranial tumor types remain that have not yet been assessed for BRAF V600E mutation status or the number of cases analyzed in previous studies was restricted to a few samples. Since the B-raf kinase inhibitor therapy poses a potential effective treatment option, a wide screening for mutations in different tumor entities is essential to determine tumor types amenable to treatment with targeted therapies. Therefore a relatively large number of gliosarcomas, meningiomas, choroid plexus tumors and non-melanoma brain metastases were included and analyzed in our cohort.

The role of BRAF mutations in primary central nervous system tumors has been addressed by several studies that mostly showed that the aberration is a rare event in gliomas. In glioblastoma the mutation frequency in the literature ranges from 2–6 %. The few patients reported with mutated glioblastomas were exceptionally young [21, 39–41]. No mutated grade II or III glioma has been found in these studies and unfortunately, the detailed histomorphology of mutated glioblastomas was not described. In our series the frequency with less than 1 % (3/312) was lower than previously reported. Combining the data from all four studies including the current one, out of 505 glioblastomas analyzed in total only 8 were BRAF V600E mutated (1.5 % of all tumors classified as glioblastomas WHO grade IV).

Interestingly, all of our three immunopositive cases showed epithelioid features and two cases were exceptionally young at the time of diagnosis. It has been reported that the rare epithelioid glioblastoma might be related to PXAs and harbor the BRAF V600E mutation at similar frequency (~50 % of the cases). Furthermore BRAF V600E mutated gliomas are almost exclusively seen in the pediatric population [21, 39].

This combined data indicates that all glioblastomas with epithelioid morphology or occurring below the age of 30 years may carry V600E mutations. As shown above, patients suffering from an astrocytoma with a BRAF V600E mutation are significantly younger at the time of diagnosis and using the upper 95 % confidence interval of the mutated cases, a cut off at 30 years for screening is statistically supported. Further studies are necessary to allow deeper insights into the gliomagenesis of this special tumor entity and their relation to PXA. Consistent with the previous data from Schindler et al. and Gierke et al. we did not find any V600E positive ependymal or choroid plexus tumor in 133 samples analyzed, indicating that V600E mutations are absent in these neoplasms [21, 29].

Data regarding BRAF analysis in meningiomas is limited. Schindler et al. previously analyzed 75 meningiomas without evidence for a V600E mutation [21]. Of our 117 meningeal tumors, only one case harbored a BRAF V600E mutation. This case was one of three rhabdoid meningiomas WHO grade III and of exceptionally young age (15 compared to 45 and 47 years). A pleomorphic xanthoastrocytoma or other potential differential diagnoses were ruled out by extensive immunohistochemistry reworking of the cases. All other anaplastic meningiomas WHO grade III of this cohort showed no evidence of V600E mutation (0/24). Rhabdoid meningiomas may also display some epithelial qualities of the tumor cells on histopathological examination, similar to the epithelioid glioblastoma variant, but these tumors can be easily distinguished by immunohistochemistry for epithelial membrane antigen and glial fibrillary acidic protein (GFAP) [42, 43]. Just recently, a case of a BRAF mutated, metastasizing rhabdoid meningioma in a comatose child discovered by panel sequencing was reported [44]. Treatment with a kinase inhibitor showed efficacy by clinical improvement. There is growing evidence that BRAF V600E mutated meningeal tumors may respond to this selective inhibitor therapy [33]. This underlines the importance of BRAF mutation testing for high-grade meningiomas, especially when rhabdoid features are present. Consistent with the previous data from Schindler et al. who analyzed 14 schwannomas, we also found no mutation in 50 vestibular schwannomas, indicating that BRAF mutations are absent or very rare in these tumors [21].

Among the cerebral metastases, a total of 29 out of 135 tumors were BRAF V600E mutated and detected by the mutation-specific VE-1 antibody. We found a mutation frequency in cerebral melanoma metastases of 41.38 % (24/58), which is in line with the incidence reported in the literature (55.3 %) [13]. The mutation rate in papillary thyroid carcinomas has been described to be around 45 % [17, 18]. Capper et al. showed a slightly lower rate in cerebral metastases with 33 % [13]. We assessed the only one metastatic case in our cohort, which was BRAF V600E mutated.

Interestingly, we found four weakly immunopositive breast cancer metastases, which were all of wildtype status after sequencing (overall 0/22). This data is in line with other studies on cerebral metastases that suggested absence of this mutation in metastasized breast cancer (in 117 [13] and seven cases [15]). Additionally, two non-small cell lung cancer (NSCLC) adenocarcinoma metastases were immunopositive but did not show a BRAF V600E mutation in the sequencing analysis. In NSCLC the mutation rate for primary tumors was described as 4 % in a large study by Cardarella et al. [14]. Villalva et al. could not find a single mutated NSCLC cerebral metastasis in 77 cases [15], while Capper et al. showed only one out of 355 NSCLCs to be mutated. Out of these 355 cases there were 169 adenocarcinomas, which were all immunonegative [13]. All 29 NSCLSs of our study were adenocarcinomas and were immunonegative, in line with previous data.

We have no explanation for the false immunopositivity of these cases, but we were able to rule out sequencing failure since all samples had at least 80 % tumor content. Although the mutation-specific antibody has been successfully validated in several types of cancer [6–11], false-positive staining with the VE-1 antibody has been reported previously for pituitary adenomas [45]. In this case, the positive VE-1 staining was confirmed by independent laboratories but direct sequencing of BRAF and the putative homologs A-Raf proto-oncogene serine/threonine protein kinase (ARAF) and Raf-1 proto-oncogene serine/threonine protein kinase (CRAF) ruled out cross reactivity [45]. It is likely that a similar cross reactivity to an unknown homolog may exist in breast adenocarcinomas although false-positive staining was weaker compared to some of the pituitary adenomas. Our data suggest that, while VE-1 may represent a specific tool for detecting BRAF V600E mutations in validated tumors, such as melanoma or colorectal cancer metastases, it is unsuitable for detecting potential BRAF V600E mutations in breast cancer, lung adenocarcinomas and pituitary adenomas, further supporting that the specificity of VE-1 should be thoroughly examined for each tumor entity by paralleled genetic mutation analysis prior to routine application for research or diagnostics.

The mutation rate in primary colorectal carcinomas has been described in a large study by Roth et al. with 7.9 % [46]. A different study focusing on colorectal cancer reported up to 13 % BRAF V600E mutated tumors [47]. Capper et al. were able to reveal 5.5 % of cerebral metastases to be mutated as well (4/72 [13]). Just as reported by Villalva et al. in 2013, we found two out of four cerebral metastases to be immunopositive for the BRAF V600E mutation [15]. It is possible, that the rate may be exceptionally high due to our small sample size. Further studies with larger numbers of colorectal cancer brain metastases are needed to determine the actual frequency of V600E mutations in these tumors.

One cerebral metastasis of a hepatocellular carcinoma was also included in this study and was immunopositive for BRAF V600E (confirmed by sequencing). In the analysis by Capper et al. five cerebral metastases of HCC were all immunonegative [13]. The BRAF V600E mutation has been described in a series of Italian hepatocellular carcinomas and discussed as one of the driving mutations in carcinogenesis [48]. The kinase inhibitor sorafenib is an established targeted therapy option in hepatocellular carcinoma in loco typico and has been approved by the American Food and Drug Administration (FDA) [49, 50]. Our case is the first BRAF mutated cerebral metastasis of a hepatocellular carcinoma that has been reported in the literature. To our best knowledge, there are no reports of hepatocellular carcinomas treated with BRAF specific kinase inhibitors such as vemurafenib or dabrafenib.

No BRAF V600E mutation was found in a series of 534 gastroesophageal tumors by Preusser et al. [51]. Three cases of another series were also negative [13]. In contrary to these findings, one out of two assessed esophageal adenocarcinoma brain metastases of this study was mutated. The tumor origin was verified via strong immunopositivity for CDX2 (Caudal type homeobox 2, a marker for intestinal epithelial cells) in both cases and review of primary tumors. This data indicates that V600E mutations are not restricted to the lower intestinal tract and all brain metastases with CK20 (Cytokeratin 20, a marker for intestinal epithelial cells) and CDX2 immunoprofile should be examined for a possible V600E mutation.

### Limitations of this study

The tissue microarrays were constructed by selecting representative tumor cores excluding necrotic tumor areas, inflammation, stroma-rich areas or infiltration borders. In most cases two donor cylinders were obtained to assess potential morphologic heterogeneity of tumors and aligned on recipient blocks. Instead of using the more commonly used smaller 600 μm sized punches we used 1000 μm and, when possible, 2000 μm sized punches in our study to obtain more available tumor tissue for staining examination. We did not observe a heterogenous staining in tumor areas of mutated samples. Together with the rather large size of the samples and the confirmation of wildtype sequencing in selected negative samples a false negative staining is highly unlikely but remains possible. According to our study design, only VE-1 positive cases were sequenced, so the rate of potential false-negative cases was not assessed with this method. Besides the reported four false-positive breast cancer and two NSCLC adenocarcinoma metastases, only one gliosarcoma showed very weak immunopositivity of the mesenchymal component and was of wildtype status after Sanger sequencing control. Those tumors that were mutated and confirmed via sequencing usually had moderate to strong staining signal in all tumor cells available on the TMA sample.

A high correlation of immunohistochemical results of the VE-1 antibody with DNA-sequencing has been shown recently by Dvorak and colleagues. A sensitivity of 98.6 % and a specificity of 99.1 % were demonstrated in colorectal and papillary thyroid carcinomas [8]. Similar rates were described before in primary lung adenocarcinomas [9], malignant melanomas [7] and pleomorphic xanthoastrocytomas [10].

However, technical problems with the VE-1 antibody have been reported. Several studies revealed that the antibody is not reliable for the assessment of pituitary adenomas. It stained normal anterior pituitary tissue, while no BRAF mutated case was detected via sequencing controls [6, 45, 52, 53]. In colorectal carcinoma the antibody showed no satisfactory correlation regarding routine usage [54, 55]. A good correlation between the VE-1 antibody immunohistochemistry and sequencing for BRAF V600E mutations has been reported in brain metastases [13]. Kleinschmidt-DeMasters et al. experienced good reliability of immunohistochemistry in gliomas compared to sequencing and also reported of homogenous staining. Only unique cases with older tissue samples displayed some heterogeneous areas and even staining intensity variability from cell to cell [37]. Positive antibody staining in gliomas can be directly attributed to neoplastic cells within non-neoplastic background neuropil [12]. We experienced only homogenous staining in our samples. A high staining specificity was observed in three glioblastomas and the positive meningioma.

### Screening recommendations

Immunohistochemical screening should be considered in selected cases, followed by sequencing validation in tumor types with limited data on sensitivity and specificity or in metastases for which the origin is not entirely clear. We consider VE-1 immunostaining as a useful first step in tumor samples to elucidate potential candidates to be sequenced in the next step, especially epithelioid glioblastomas, astrocytic tumors occurring below 30 years of age and rhabdoid meningiomas. In tumor types with sufficient validation data on immunohistochemistry and sequencing available, i.e. melanoma, colorectal and thyroid carcinoma and pleomorphic xanthoastrocytomas, VE-1 immunostaining might be sufficient to indicate BRAF V600E targeted therapy.

## Conclusion

The BRAF V600E mutation can enable a potentially effective targeted therapy in cerebral neoplasms. Thus immunohistochemical screening should be considered in selected cases. For neoplasms where high rates of sensitivity and specificity of VE-1 immunostaining have been shown previously (colorectal carcinoma, thyroid cancer, malignant melanoma, glioblastoma and pleomorphic xanthoastrocytomas) BRAF V600E immunostaining is sufficient for screening purposes. We also recommend routine immunohistochemical staining followed by sequencing validation in rare CNS metastases or metastases of unknown primary. Among brain tumors, besides the already established entities such as pleomophic xanthoastrocytoma and ganglioglioma, especially epithelioid glioblastomas, astrocytic tumors occurring before the age of 30 years as well as rhabdoid meningiomas should undergo routine BRAF V600E mutation analysis. Since the potential of the targeted therapy with kinase inhibitors has been shown in several exemplary case reports, the therapeutic role of a BRAF targeted treatment in these tumors needs further assessment. Immunohistochemical analysis using mutation-specific VE-1 antibody on tissue microarrays is a feasible, time-efficient and cost-efficient approach to high-throughput screening for BRAF V600E mutations in large brain tumor series and metastases.

## Abbreviations

ARAF, Protooncogene encoding the serine/threonine-protein kinase A-Raf; BRAF, Protooncogene encoding the serine/threonine-protein kinase B-Raf; CDX2, Gene of the homeobox protein CDX-2, an intestinal epithelial marker; CK20, Cytokeratin 20, a protein in entereocytes; CNS, Central nervous system; CRAF, Protooncogene encoding the serine/threonine-protein kinase C-Raf; DNA, Deoxyribonucleic acid; FDA, Food and Drug Administration; GFAP, Glial fibrillary acidic protein, an intermediate filament in the central nervous system; H&E, Hematoxylin and eosin, a standard stain in histopathological tumor assessment; HCC, Hepatocellular carcinoma; KIAA1549, Gene regularly found to be fused to BRAF in pilocytic astrocytomas; MRI, Magnetic resonance imaging; NSCLC, Non-small cell lung cancer; PCR, Polymerase chain reaction; PXA, Pleomorphic xanthoastrocytoma; TMA, Tissue microarray; VE-1, Murine monoclonal antibody targeting the BRAF V600E mutation

## Additional file

Additional file 1: List of all analyzed tumor samples with the corresponding histopathological diagnosis and age atdiagnosis. (XLS 129 kb)
